# Supporting Self-management and Quality of Life in Bipolar Disorder With the PolarUs App (Alpha): Protocol for a Mixed Methods Study

**DOI:** 10.2196/36213

**Published:** 2022-08-04

**Authors:** Erin E Michalak, Steven J Barnes, Emma Morton, Heather L O'Brien, Greg Murray, Rachelle Hole, Denny Meyer

**Affiliations:** 1 Department of Psychiatry University of British Columbia Vancouver, BC Canada; 2 Department of Psychology University of British Columbia Vancouver, BC Canada; 3 School of Information University of British Columbia Vancouver, BC Canada; 4 Centre for Mental Health Swinburne University of Technology Melbourne, BC Canada; 5 Canadian Institute for Inclusion and Citizenship University of British Columbia Kelowna, BC Canada

**Keywords:** eHealth, mobile health, mHealth, bipolar disorder, self-management, engagement, mobile phone

## Abstract

**Background:**

Quality of life (QoL) is increasingly being recognized as a key outcome of interventions for bipolar disorder (BD). Mobile phone apps can increase access to evidence-based self-management strategies and provide real-time support. However, although individuals with lived experiences desire support with monitoring and improving broader health domains, existing BD apps largely target mood symptoms only. Further, evidence from the broader mobile health (mHealth) literature has shown that the desires and goals of end users are not adequately considered during app development, and as a result, engagement with mental health apps is suboptimal. To capitalize on the potential of apps to optimize wellness in BD, there is a need for interventions developed in consultation with real-world users designed to support QoL self-monitoring and self-management.

**Objective:**

This mixed methods pilot study was designed to evaluate the alpha version of the newly developed PolarUs app, developed to support QoL self-monitoring and self-management in people with BD. Co-designed using a community-based participatory research framework, the PolarUs app builds on the web-based adaptation of a BD-specific QoL self-assessment measure and integrates material from a web-based portal providing information on evidence-informed self-management strategies in BD. The primary objectives of this project were to evaluate PolarUs app feasibility (via behavioral use metrics), the impact of PolarUs (via the Brief Quality of Life in Bipolar Disorder scale, our primary outcome measure), and explore engagement with the PolarUs app (via quantitative and qualitative methods).

**Methods:**

Participants will be residents of North America (N=150), aged >18 years, with a Diagnostic and Statistical Manual of Mental Disorders, Fourth Edition, Text Revision diagnosis of BD type 1, BD type 2, or BD not otherwise specified as assessed by structured diagnostic interview. An embedded mixed methods research design will be adopted; qualitative interviews with a purposefully selected subsample (approximately, n=30) of participants will be conducted to explore in more depth feasibility, impact, and engagement with the PolarUs app over the 12-week study period.

**Results:**

At the time of publication of this protocol, the development of the alpha version of the PolarUs app was complete. Participant enrollment has begun in June 2022. Data collection is expected to be completed by December 2022.

**Conclusions:**

Beyond contributing knowledge on the feasibility and impact of a novel app to support QoL and self-management in BD, this study will also provide new insights related to engagement with mHealth apps. Furthermore, it will function as a case study of successful co-design between people with BD, health care providers, and BD researchers, providing a template for the future use of community-based participatory research frameworks in mHealth intervention development. The results will be used to further refine the PolarUs app and inform the design of a larger clinical trial.

**International Registered Report Identifier (IRRID):**

PRR1-10.2196/36213

## Introduction

### Background

Bipolar disorder (BD) is characterized by episodes of pathologically depressed or elevated mood states, with a global estimated lifetime prevalence of 2.4% [1]. Although BD can be associated with significant distress, disability, and mortality [2], many individuals with BD report a good quality of life (QoL) [3,4]. Accordingly, optimal treatment of BD involves not only symptom management but also attention to QoL [5,6]. For example, there is only a small to moderate correlation between mood stability and QoL [7-10], indicating that interventions *specifically* targeting QoL are required.

QoL is a treatment outcome prioritized by people living with BD [11-13] and encompasses a broad range of constructs from symptoms and functional impacts to well-being and satisfaction with life domains [6,14]. People with BD have highlighted the importance of life areas directly affected by BD (mood, sleep, physical health, and cognition), functioning and participation (home, work, education, leisure, finances, and relationships), and subjective experiences (self-esteem, spirituality, identity, and independence), which form the basis of BD-specific QoL assessment [14,15]. Self-management interventions align well with QoL-oriented treatment frameworks by assisting an individual with the process of monitoring, responding to, and coping with the impacts of BD [16]. Although psychoeducation about self-management strategies is increasingly incorporated into BD treatment guidelines [17-19], there are substantive barriers to accessing such support. Only 50% of patients in treatment for BD receive psychosocial services such as psychoeducation [20], and people with BD report receiving inadequate knowledge of QoL-focused self-management strategies [21]. Stigma and mistrust in the health care system may also discourage seeking psychoeducation about self-management [16,22]. Unfortunately, the COVID-19 pandemic has introduced additional barriers to obtaining psychosocial interventions [23,24].

Digital health interventions have been suggested as a means to improve access to self-management information and support [16,25]. A total of 2 innovative digital health projects were recently developed with a focus on optimizing QoL in BD. First, only the BD-specific QoL instrument (Quality of Life in Bipolar Disorder [QoL.BD] scale [14]) was adapted to a web-based format (the QoL Tool [26]). The QoL Tool has comparable psychometric properties with those of the pen-and-paper version [27], and individuals with BD reported positive experiences using the web-based format [28]. Second, a novel web-based suite of multimedia evidence–informed self-management tools for people with BD (the Bipolar Wellness Centre [29]) was developed as a partner website to the QoL Tool and has been found to enhance both subjective recovery and QoL [30]. Although these websites have each produced positive impact, they are not integrated, and unlike smartphone apps are not able to provide *in the moment* responsive support. Moreover, mobile app–based self-management and self-monitoring interventions have been shown to have higher levels of engagement than analogous web-based versions [31].

Interest in and use of apps to address mental health needs is high among people with BD. A recent survey found that 93% of people with BD own smartphones, and 77% expressed willingness to receive support with self-management strategies via an app [32]. However, to capitalize on the potential of app-based interventions for BD, the field must address the pressing challenge of user engagement and retention [33]. Evidence from the broader field of digital mental health research suggests that user interest does not necessarily translate to sustained use of web- or app-based interventions. For example, clinical trials of apps for depression have demonstrated high dropout rates [34], and data on publicly available digital self-help interventions for depression or anxiety suggest that program completion and long-term use are rare [35].

Although engagement with mobile apps for BD has not been widely formally evaluated, a qualitative analysis of app store reviews highlighted several unmet user needs related to features and content [36]. QoL-focused app-based interventions have the potential to increase engagement and retention by addressing some of these unmet needs for 3 reasons. First, given the breadth of the QoL construct, such interventions could address the full spectrum of life domains that individuals with BD nominate as important foci for self-management [37-40]. Second, in contrast to traditional symptom-focused monitoring, which tends to highlight dysfunction and can sometimes increase depressive symptoms in BD [41], QoL-focused self-monitoring may help draw attention to personal strengths [28]. Third, as the QoL framework aligns with the treatment goals of many patients, therapeutic alliance and motivation to engage in treatment may be enhanced by a QoL-focused app [42,43]. Indeed, individuals with BD have described participating in a QoL-focused intervention as empowering [21] and that QoL self-monitoring encourages behavior change [28].

The alpha version of the app that will be evaluated in this study, the PolarUs app, builds on and advances a decade of research by the Collaborative Research Team to Study Psychosocial Issues in Bipolar Disorder (CREST.BD). Specifically, the PolarUs app synthesizes and advances the evidence and resources contained in the QoL Tool and Bipolar Wellness Centre and incorporates additional features and content nominated as important by individuals with BD [33]. Furthermore, individuals with BD and BD researchers co-designed the PolarUs app, which incorporates evidence-informed self-management strategies. Although human or peer support has been shown to enhance engagement with digital health interventions [44,45], this comes with a trade-off in terms of feasibility, particularly in terms of scale-up. Unfortunately, research-led interventions are rarely publicly available [46,47], and researchers are increasingly encouraged to formulate plans for sustainable dissemination beyond the clinical trial [48]. Although the PolarUs app can be used to facilitate or support one-to-one therapy, it has primarily been designed for use as a self-guided program. In this paper, we describe the procedures to be used in a mixed methods study that will explore the feasibility and impact of the PolarUs app and allow for an exploration of patterns of engagement with the app.

### Objectives and Hypotheses

There are 3 overarching objectives for this pilot study.

#### Objective 1: Evaluating PolarUs App Feasibility

To explore feasibility, we will assess rates of adherence and use of the alpha version of the PolarUs app over a 12-week study period.

#### Objective 2: Evaluating PolarUs App Impact

To evaluate the impact of the alpha version of the PolarUs app, we will assess QoL (using the Brief QoL.BD, our primary outcome measure) over the 12-week study period; we hypothesize that QoL will improve over that time. In addition, we will explore the impact of the app on our secondary outcome measures (ie, mood symptoms, self-efficacy in illness management, subjective recovery, and self-compassion).

#### Objective 3: Exploring PolarUs App Engagement

Given the relative immaturity of methods for measuring mobile health (mHealth) app engagement (*Discussion* section), we will apply a mixed methods approach to more deeply explore patterns of engagement with the PolarUs app.

## Methods

### Design

#### Overview

A sample of 150 adult research participants with a confirmed diagnosis of BD will be recruited to evaluate the alpha version of the PolarUs app. Our chosen assessment period for the evaluation of the PolarUs app is 12 weeks. Most app evaluation studies are 4 to 8 weeks [49], but this may be an insufficient time period within which to assess trajectories of change in self-management behaviors, QoL, or app engagement.

A mixed methods research approach [50] is embedded in the design of this study. Qualitative methods are added to a traditional quantitative design as a single approach, and data set will not be adequate to successfully address our 3 objectives. In the case of this research, qualitative interviews with a purposefully selected subsample (approximately, n=30) of participants will be conducted to explore in more depth feasibility, impact, and engagement with the PolarUs app over the 12-week study period. Qualitative methods are used to enhance our understanding of the quantitative results, improving the overall design through these complementary approaches [50].

#### Co-design of the PolarUs App

CREST.BD specializes in community-based participatory research, where researchers and knowledge users (in this case, people with BD, their supporters, and BD health care providers) work hand in hand [51]. Lived experiences of BD and co-design methods were integrated into all aspects of PolarUs app development and the design of the evaluation study. For example, the colead principal investigator of the project (SJB) lives with BD, as do some of the coinvestigators. Moreover, the app and study design are guided by a 7-member advisory group, which meets approximately monthly. All members of the advisory group have lived experience of BD as well as a diverse array of additional expertise, including user interface design, interactivity, graphic design, writing, and patient-engaged research.

In addition to our co-design approach, input from a broader international community of individuals living with BD was gathered through a survey. This survey was used to solicit community perspectives on features and content deemed important for inclusion in a BD-specific mHealth app [33].

#### Functionality of the PolarUs App

**Figure 1:**
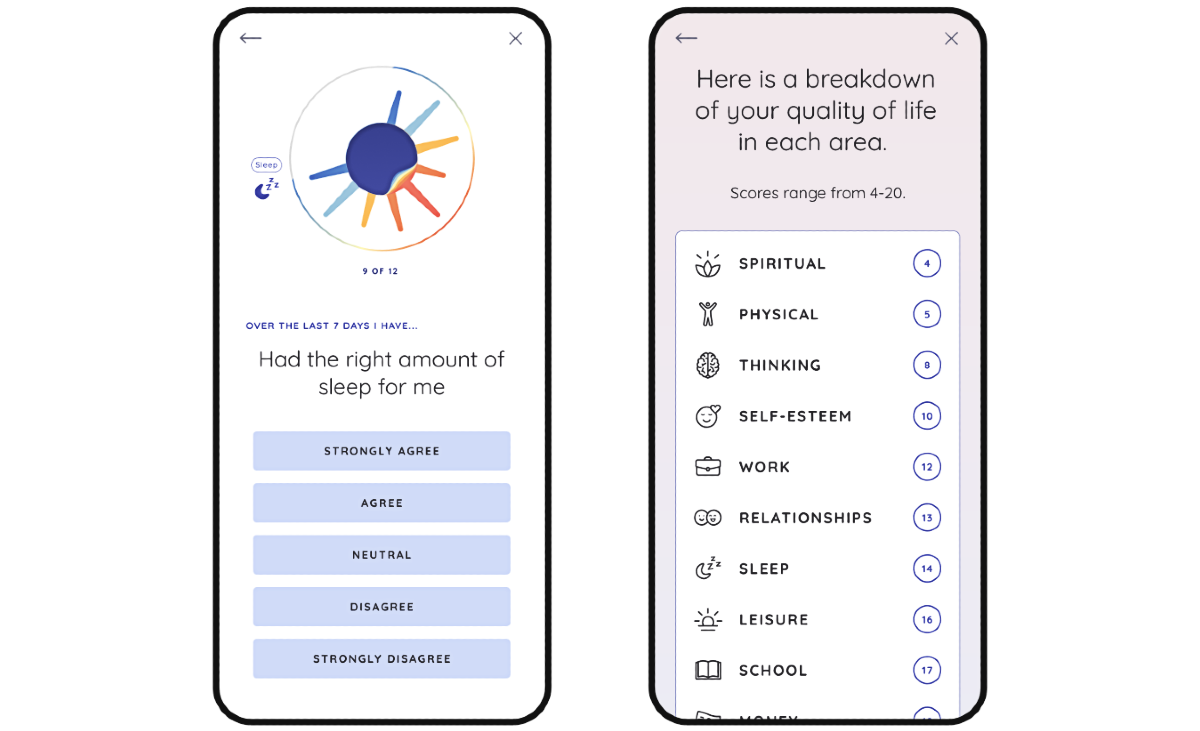
PolarUs app users will complete a self-assessment using the Quality of Life in Bipolar Disorder (QoL.BD) scale at the end of each week and at the end of each month. The image on the left illustrates the presentation of a question from the QoL.BD. The image on the right illustrates a summary screen that is presented to users once they complete the QoL.BD.

**Figure 2:**
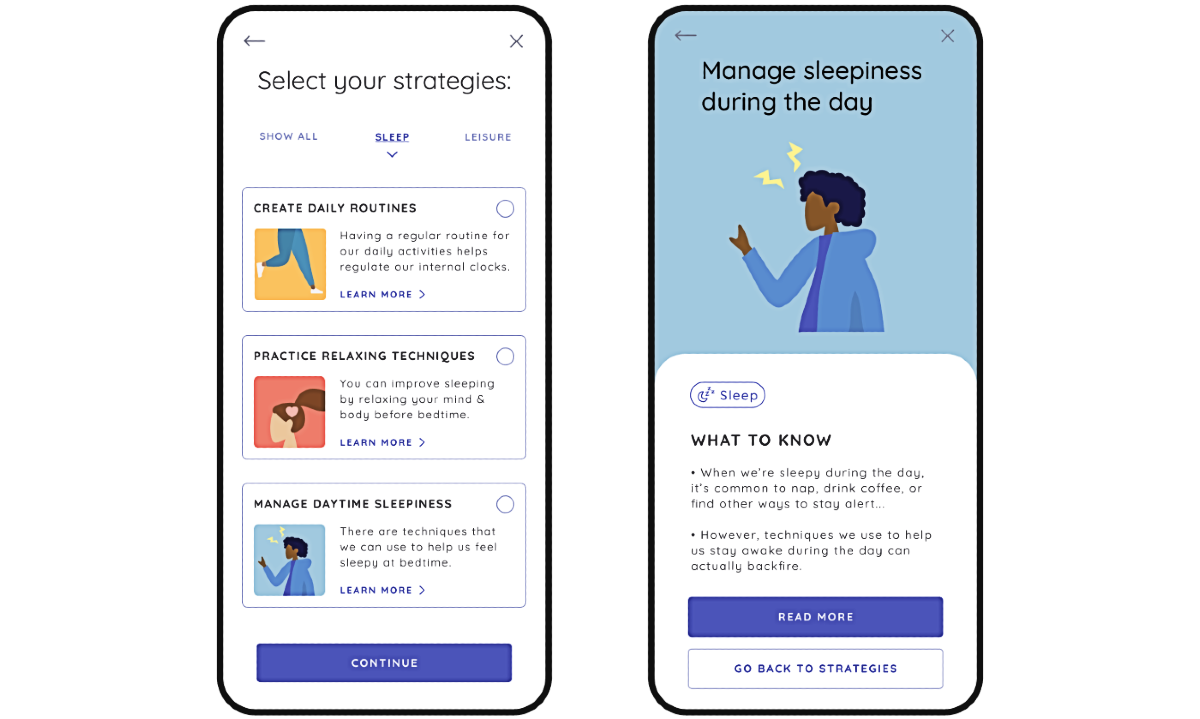
Participants will be prompted (eg, “How are you feeling today?”) to complete the Quality of Life in Bipolar Disorder and other self-monitoring activities at regular intervals during the 12-week study. The image on the left illustrates a prompt to do a daily mood check-in. The image on the right illustrates how users can view the history of their Quality of Life in Bipolar Disorder (QoL.BD) scores over the 12-week period of the study. Participants can peruse any one of many resources that might help them with the self-management strategies they are currently using at any time (eg, bottom portion of left image).

The PolarUs app incorporates and expands upon the evidence, resources, and tools currently provided in CREST.BD’s Bipolar Wellness Centre [29] and the QoL Tool [26]. As with the Bipolar Wellness Centre, content in the PolarUs app is organized according to the 14 life areas assessed by the QoL.BD. After completing a QoL.BD assessment (Figure 1) at baseline, users will be able to select up to 3 QoL.BD life areas they would like to improve, after which they can select up to 4 relevant self-management strategies to implement over a subsequent 4-week period. During each 4-week period of this 12-week study, users are encouraged to self-monitor their QoL, mood, and sleep at regular intervals (Figure 2). Users receive encouragement while using the app in various forms, including notifications and some types of gamification.

### Typical Use Scenario for the PolarUs App in This Study

**Figure 3:**
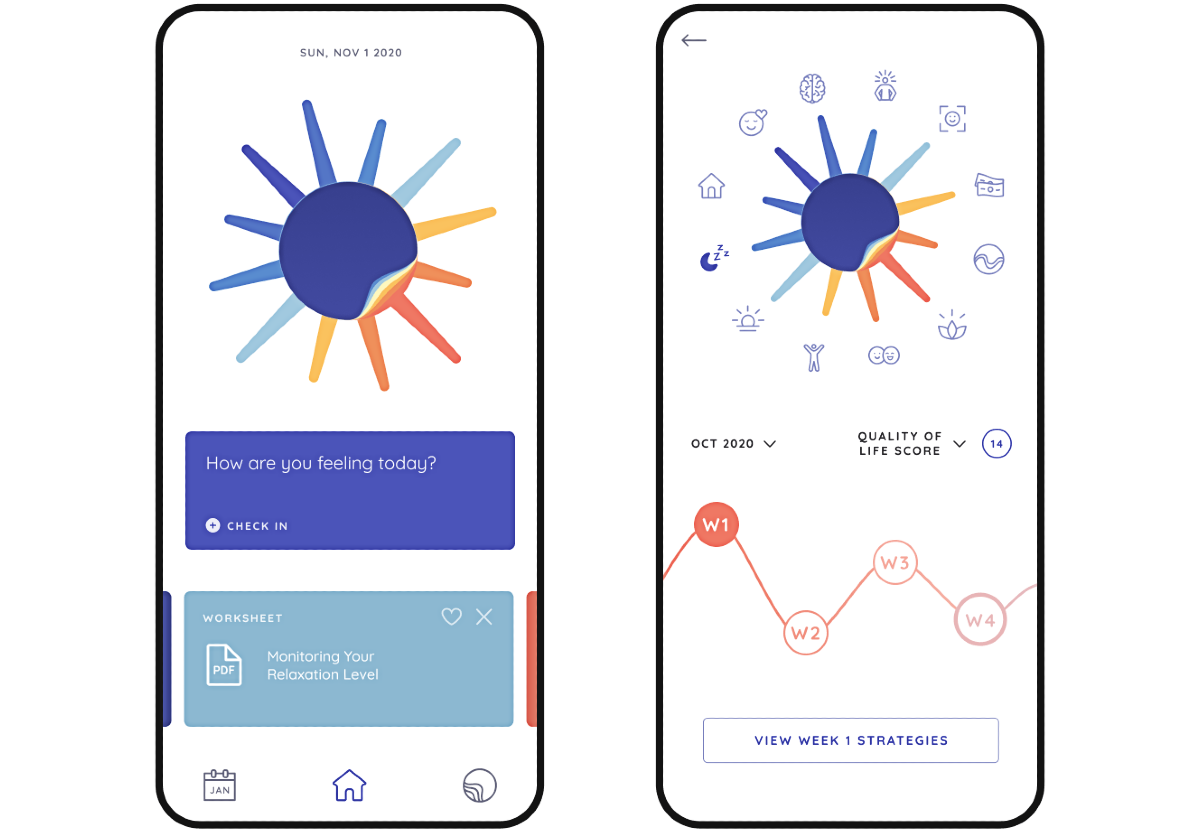
The PolarUs app contains information on evidence-informed self-management strategies for each Quality of Life in Bipolar Disorder (QoL.BD) life area. This figure shows 2 screens from the PolarUs app: one with information about the sleep life area (right image) and one with a list of strategies for improving the sleep life area that a user can choose to focus on.

A typical PolarUs user will engage in the following in-app activities during the course of this study, in the following sequence:

1. Users begin by completing an in-app baseline full QoL.BD assessment (Figure 1).
2. Users then have the opportunity to review their baseline QoL.BD assessment (right image in Figure 2).
3. Users are then prompted to choose up to 3 QoL.BD life areas to focus on over the next 4 weeks.
4. Users are then prompted to choose up to 4 relevant self-management strategies to use over the next 4 weeks (Figure 3).
5. Users are then encouraged to engage in those self-management strategies over the next 4 weeks. They are free to change their self-management strategies over the course of the 4-week period.
6. Once they select their self-management strategies, users are provided with a list of resources related to each strategy that they can review at their leisure.
7. Users are prompted to perform a daily *check-in* regarding their sleep quality and mood (left image in Figure 2).
8. After each period of 7 days, users are prompted to complete the Brief QoL.BD. They can review all their QoL.BD data at any time by navigating to the history screens (eg, right image in Figure 2).
9. At the end of each 4-week period, users are prompted to complete the full QoL.BD, and then steps 3 to 9 are repeated until the 12-week period of this study ends.

### Technical Specifications of the Alpha PolarUs App

**Figure 4:**
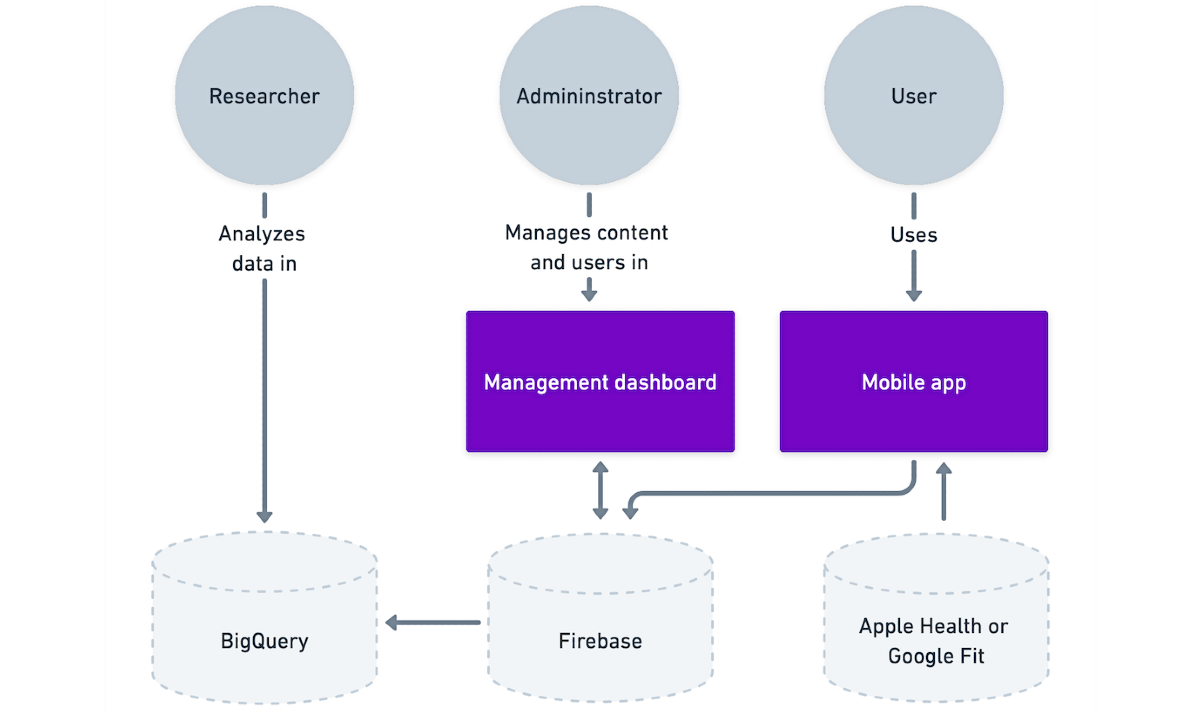
PolarUs app architecture.

The alpha version of the PolarUs app operates on either the Android or iOS smartphone platform. It was built using the open-source Maslo platform (maslo.ai; [52]), which incorporates several technologies. For its frontend, the Maslo platform uses React Native [53], Three.js [54], and Javascript or Typescript. For its backend, the Maslo platform uses Firebase. In addition to using the Maslo platform, the PolarUs app uses Neo4J [55] as its graph database. The architecture of the PolarUs app is illustrated in Figure 4.

Recruitment notices will be circulated to CREST.BD partner organizations and promoted on multiple CREST.BD social media platforms (eg, Facebook, Twitter, and Instagram), on the CREST.BD website [56], and via a project-specific landing page [57]. Participants will be residents of North America, aged >18 years, with a primary Diagnostic and Statistical Manual of Mental Disorders, Fourth Edition, Text Revision diagnosis of BD type 1, BD type 1, or BD not otherwise specified as assessed by the Mini-International Neuropsychiatric Interview version 7.0 [58]. Minimally restrictive diagnostic exclusion criteria will be set to aid generalizability; those currently experiencing psychosis and those with active suicidal ideation as assessed by diagnostic interviews will be excluded [58]. Participants will also need to be smartphone users, agree to install the app, agree to receive notifications from the app, and have sufficient understanding of written and spoken English to provide informed consent and engage with the app. In addition, participants will, for the purposes of the research study, have the option of consenting to the sharing of their health and behavioral data from the PolarUs app and from the Apple Health or Google Fit apps on their smartphone. Apple Health and Google Fit will not have access to the data unless the user gives permission to share their data with Apple or Google independent of the app. Participants will be invited to consent separately to engage with the qualitative arm of the study.

### Ethics Approval

Ethics approval for the study has been granted by the University of British Columbia Behavioural Research Ethics Board office (H21-02042).

### Assessment and Data Collection Procedures

#### Overview

Table 1 summarizes the assessment procedures used. The Mini-International Neuropsychiatric Interview version 7.0 will be administered by experienced research assistants at baseline via the Zoom teleconferencing platform or telephone to confirm diagnostic eligibility (ie, a Diagnostic and Statistical Manual of Mental Disorders, Fourth Edition, Text Revision diagnosis of BD type 1, BD type 2, or BD not otherwise specified) and identify any comorbid diagnoses [58]. This structured baseline interview will also be used to record sociodemographic variables and conduct the objective mood rating scales.

Clinician-administered and self-report scales (see *In-app QoL.BD: Primary Outcome Measure*, *Clinician-Administered Scales,* and *Qualtrics-Administered Self-Report Scales* sections) will be completed at baseline and at monthly intervals during the 12-week pilot study. Most self-report measures will be conducted via a secure, encrypted web-based survey platform (ie, UBC Qualtrics) that stores its data on Canadian servers. Research assistants (Caden Poh, Bryn Manns, and Priya Johal) will monitor the completion of Qualtrics questionnaires on a regular basis and contact participants to facilitate completion when such support may be required.

Some self-report measures (eg, QoL.BD) will be administered within the app. App use data will be automatically collected in real time. All data collected by the PolarUs app will be transmitted using end-to-end encryption to a secure database on a Canadian server.

At the end of the 12-week evaluation period, a subset of participants will be invited to participate in qualitative interviews. The assessment procedures and instruments to be used in this study are described below, in relation to our study objectives.

**Table 1:** Summary of self-report, clinician-administered, behavioral, and qualitative data to be collected.

Data type	Outcomes	Scale	Delivery method (frequency)
Self-report	Condition-specific QoL^a^ General QoL Chronic Disease Self-Efficacy Mood Personal recovery Self-compassion Subjective app engagement App acceptability	Full QoL.BD^b^ Brief QoL.BD WHOQOL-BREF^c^ Stanford’s Chronic Disease Self-Efficacy: “Manage Disease in General” subscale Positive and Negative Affect Schedule Bipolar Recovery Questionnaire Self-Compassion Scale-Short Form User Engagement Scale-Short Form Mobile App Rating Scale (user version)	In-app (monthly) In-app (weekly) Qualtrics (monthly) Qualtrics (monthly) Qualtrics (monthly) Qualtrics (monthly) Qualtrics (monthly) Qualtrics (monthly) Qualtrics (first month)
Behavioral	Adherence (number of log-ins per week and number of Brief QoL.BD completed) App use (number and frequency of pages accessed; time spent on the app per session and overall; time spent on specific pages; number and length of unique sessions; length of time between unique sessions) Behavioral health data (eg, daily activity, heart rate, sleep data, and nutrition)	N/A^d^	In-app (real-time) In-app (real-time) Apple health (iOS) or Google Fit (Android)
Clinician-administered	Diagnosis Depressive symptoms Manic symptoms	MINI^e^ Montgomery-Asberg Depression Rating Scale Young Mania Rating Scale	Telephone (baseline) Telephone (monthly) Telephone (monthly)
Qualitative	Subjective app engagement and impacts	N/A	Telephone or Zoom (after intervention)

^a^QoL: quality of life.

^b^QoL.BD: Quality of Life in Bipolar Disorder.

^c^WHOQOL-BREF: Brief World Health Organization Quality of Life.

^d^N/A: not applicable.

^e^MINI: Mini-International Neuropsychiatric Interview.

#### Objective 1: Evaluating PolarUs App Adherence

Our first objective will be to evaluate levels of adherence with and use of the alpha version of the PolarUs app over the 12-week study period. For the purposes of this study, adherence will be defined as beginning to use the app and continuing to do so in a prescribed manner; adherence is rooted in use behaviors (eg, frequency and duration of app or specific app feature use) as opposed to subjective experience [59].

Key use behaviors will be assessed primarily by the number of weekly QoL.BD questionnaires completed by participants over the 12-week study period. These use data will be used to produce a profile for each participant for each 4-week period. Then, those participant use profiles will be used to classify participants into use clusters, such as *regularly used*, *intermittently used*, and *initially used*, to explore use patterns associated with use of the PolarUs app. Additional use data will also be collected, including the number and frequency of the QoL domain and self-management strategy content pages accessed, time spent in the app, both per-session and overall time spent on a specific domain and self-management strategy content pages, number and length of unique sessions, and length of time between unique sessions.

#### Objective 2: Evaluating PolarUs App Impact

##### Overview

Our second objective will be to evaluate the impact of the alpha version of the PolarUs app for improving QoL, as measured weekly across the 12-week period of the study using the in-app Brief QoL.BD—our primary measure of impact. Exploratory analyses will examine whether the app affects our secondary outcome measures, as assessed by clinician-administered and self-report scales: mood symptoms, self-efficacy in illness management, subjective recovery, and self-compassion (described below).

##### In-App QoL.BD: Primary Outcome Measure

The QoL.BD is the first and to date only instrument developed to specifically assess QoL in terms of the life areas prioritized by individuals living with BD [14]. QoL.BD items were derived from interviews with people with lived experience of BD [15], health care providers, and BD subject matter experts, in combination with a comprehensive literature review.

The full 56-item QoL.BD assesses 12 core (physical, sleep, mood, cognition, leisure, social, spirituality, finance, household, self-esteem, independence, and identity) and 2 optional (work and study) life areas, each containing 4 self-report Likert scale items (1=strongly disagree to 5=strongly agree). An overall score (range 48-240) can be calculated by summing the responses to the 48 items of the 12 core domains. Higher overall scores represent greater satisfaction with life. The Brief QoL.BD is an abbreviated version of the full scale that contains 12 items representing the core domains (overall score range 12-60). During initial field testing, both versions of the QoL.BD had excellent internal reliability (Cronbach α>.8), and the Brief QoL.BD demonstrated a higher sensitivity to changes in clinician-rated symptoms of depression than generic QoL measures [14]. The QoL.BD has been used in international clinical trials, with sensitivity to treatment effects demonstrated [10]. Construct validity of the Brief and full QoL.BD has been demonstrated through associations with symptoms of mania and depression, generic QoL instruments, and functioning [10,14]. A web-based adaptation of the full instrument, the QoL Tool, has been psychometrically validated [27].

##### Clinician-Administered Scales

The 10-item Montgomery-Asberg Depression Rating Scale [60] will be administered using a structured interview guide [61]. Scores on the Montgomery-Asberg Depression Rating Scale range from 0 to 60, with higher scores indicating greater severity of depressive symptoms. Symptoms of mania will be assessed using the Young Mania Rating Scale [62], a 11-item scale with scores ranging from 0 to 60, with higher scores indicating greater severity of manic symptoms.

##### Qualtrics-Administered Self-report Scales

The Brief World Health Organization Quality of Life (WHOQOL-BREF) scale [63] will be used to assess non–BD-specific aspects of QoL (ie, domains assumed to be relevant to the general population). The WHOQOL-BREF is one of the most commonly used generic QoL instruments in the BD literature [6], and is included in addition to the QoL.BD to facilitate comparisons with other populations. This instrument is reliable and valid for populations with psychiatric illnesses [64]. The WHOQOL-BREF has 26 Likert scale items that are used to calculate QoL for 4 domains: physical, psychological, social, and environmental. Higher scores for these QoL domains (range 0-100) indicate greater life satisfaction.

Chronic Disease Self-efficacy will be assessed using the 5-item *Manage Disease in General* subscale of Stanford’s Chronic Disease Self-Efficacy Scale [65,66]. Higher scores (range 1-10) indicate greater confidence in managing the impact of a chronic health condition. Self-reported mood will be measured using the Positive and Negative Affect Schedule [67]. Higher scores on the two 10-item subscales (range 10-50) indicate greater levels of positive and negative affect.

The 36-item Bipolar Recovery Questionnaire was informed by qualitative research on the experiences of personal (as opposed to clinical) recovery in BD [68]. Higher scores (range 0-3600) indicate better self-appraised recovery. The Bipolar Recovery Questionnaire has good internal consistency and test-retest reliability and has been found to be sensitive to change in an evaluation of a cognitive behavioral therapy–based BD intervention [69].

The Self-Compassion Scale-Short Form will be used to measure self-reported self-compassion [70,71]. A total of 12 items are used to assess 6 dimensions of self-compassion: self-kindness, self-judgment, common humanity, isolation, mindfulness, and overidentification. Higher scores (range 1-5) indicate more frequent experiences of self-compassionate behaviors and attitudes.

The subjective acceptability of the PolarUs app will be assessed by using the user version of the Mobile App Rating Scale [72]. This scale contains subscales that measure attitudes toward app engagement, functionality, aesthetics, and information quality.

#### Objective 3: Exploring PolarUs App Engagement

Our third objective will apply a mixed methods approach to more deeply explore the patterns of engagement with the PolarUs app. Our quantitative scale to measure subjectively experienced engagement will be the User Engagement Scale (UES) Short Form [73,74]. Both the UES Long Form (31 items) and UES Short Form (12 items) consist of 4 subscales: focused attention (feeling absorbed in interaction with the system and losing track of time), perceived usability (negative affect experienced because of effort expended to use the system), aesthetic appeal (visual appeal of the interface), and reward (perceived benefits and interest experienced because of using the system). The UES has been used internationally by academic and industry researchers and has been found to be a reliable, valid, and sensitive measure for evaluating engagement with a range of technologies, including digital health applications [75]. Both subscale and the overall engagement scores can be calculated as the average of the included items (range 1-5), with higher scores indicating higher levels of subjective engagement with the app. The UES will be administered as a Qualtrics survey every 4 weeks and at study completion.

In-depth qualitative interviews (approximately 1 hour) will be conducted with a subsample (approximately, n=30) of participants immediately after the end of the 12-week study period. Potential interviewees will be invited primarily based on their engagement patterns (assessed quantitatively). Specifically, we will seek to capture major variations in adherence and engagement by purposeful sampling according to the individual’s use cluster (eg, *regularly used*, *intermittently used*, and *initially used*). Purposeful sampling will be used to ensure representativeness of the subsample [76] according to diversity in gender, age, ethnocultural background, and BD diagnosis.

Interviews will be semistructured, and the topics discussed will include (1) perceptions of the PolarUs app (eg, attitudes toward specific features and content), (2) experiences of engaging with the app across the intervention period, (3) facilitators and barriers to app use, and (4) subjective impacts (eg, QoL, self-management behaviors, and self-efficacy). All interviews will be conducted remotely via Zoom or telephone and will be recorded and transcribed for later analysis.

### Sample Size

To inform sample size, we benchmarked a recent meta-analysis of clinical trials of smartphone apps for depressive symptoms, which estimated dropout rates of 25% to 50% [77]. Allowing for a 33% dropout rate, 90% power, an effect size of 0.5 SD, and a nonsphericity correction of 0.7 for the final monthly QoL.BD scores for each participant, the required sample size for addressing objective 2 is estimated at 150 participants. This sample size also allows similar power levels to address the exploratory analyses related to the other measures collected monthly through Qualtrics. A challenge in estimating the sample size required is the heterogeneity in prior research that has used QoL.BD (or a variant thereof). In 2020, a search identified 13 clinical trials of psychosocial interventions in BD that reported on any QoL.BD outcomes. Significant changes in scores were uncommon, but the studies were generally limited by small sample sizes. In addition, as QoL was often used as a secondary outcome, the effect sizes were not consistently reported. However, promising effect sizes (0.4-1.42) were observed for recovery-focused cognitive behavioral therapy, dialectical behavior therapy, and web-based recovery-focused psychoeducation and mindfulness interventions [10].

Regarding our sample size for the qualitative interviews, although an approximation of sample size is informative for planning, in practice the appropriateness of the sample must be evaluated during the research process. There are no firm recommendations on the precise number of participants to include; rather, sample size is informed by attention to a number of dimensions related to the research aims, informed by pragmatic considerations and the researcher’s own experiences [78]. Given our broad aims (objectives 1-3) and our prior experiences of using qualitative methods to explore the use of digital health tools for BD [21,28], we estimate our chosen sample size of approximately 30 for the interviews to be sufficient to support meaningful thematic analyses.

### Data Management and Statistical Analyses

Responses to the Qualtrics and in-app questionnaires will be made mandatory to reduce the likelihood of missing data. Comprehensive data cleaning will occur before analyses with range and distributional checks and comparisons with published norms, where appropriate.

#### Objective 1: Examining PolarUs App Feasibility

To describe and categorize levels of adherence with and use of the PolarUs app over the 12-week study period, our behavioral measures (described above) will be used to produce a profile for each participant for each 4-week period; these profiles will then be used to classify participants into use clusters, such as *regularly used*, *intermittently used* and *initially used*. The number of categories and initial seeds for these categories will be established using an agglomerative hierarchical clustering approach (eg, Ward method) and then refined using mean values to define the participant engagement profile for each of the clusters.

#### Objective 2: Evaluating PolarUs App Impact

To provide a preliminary evaluation of the impact of the alpha version of the PolarUs app on our primary (ie, weekly Brief QoL.BD) and exploratory outcome measures (described above), we will use mixed effects modeling with random intercepts and slopes to track changes in these measures over the 12-week study period.

#### Objective 3: Exploring PolarUs App Engagement

To explore engagement patterns with the PolarUs app, two types of analyses will be used to explore the relationships between engagement and outcome trajectories of individual participants: (1) quantitative analyses of the longitudinal impact and engagement data (see above) and (2) qualitative analyses of the interview data.

In terms of the quantitative analyses, the moderating effects of participant engagement cluster on our primary and secondary measures of impact will be explored statistically by using mixed effects modeling with random intercepts and slopes and with the engagement cluster × time interaction as a fixed effect. Mixed effects modeling will allow changes in the primary and secondary measures to be explained in terms of engagement cluster membership by estimating and testing time-by-cluster interactions. The participant clusters showing the greatest benefit will be used to benchmark the best engagement behaviors over time. Mixed effects modeling will be used in the same way to assess the correlation between subjective app engagement scores and actual engagement behaviors identified with the use clusters. To appreciate the extent to which the PolarUs app is aligned with users’ self-management goals and user expectations, we will compare use data (eg, time spent) for the various self-management strategy content pages and specific QoL domains using nonparametric repeated measures analyses (eg, Friedman test). The self-management strategy content pages and specific QoL domains on which more time is spent are likely to be most closely aligned with a user’s self-management goals and expectations. All of these analyses will be further informed by qualitative analysis of the interview data.

Thematic analysis will be used for qualitative analyses [79]. Through a careful reading and rereading of the interview data, the data will be compared, contrasted, and categorized (both within and across transcripts) to identify themes. NVivo (version 12; QSR International) will be used to manage the data and facilitate data analysis.

#### Exploratory Economic Evaluation

An exploratory economic evaluation will be conducted at the end of the study, using a health care payer perspective to estimate the cost per incremental unit of QoL. These estimates will be summarized using an incremental cost-effectiveness ratio [80] and an incremental net benefit statistic [81,82]. The uncertainty of the estimates will be characterized using cost-effectiveness acceptability curves and 95% CIs [83,84].

## Results

Participant enrollment has begun in June 2022. Data collection is expected to be completed by December 2022.

## Discussion

### Overview

This protocol paper describes a pilot study designed to assess the feasibility, impact, and engagement with the alpha version of the PolarUs app for BD. This study has the following objectives: (1) to describe and categorize levels of adherence with and use of the PolarUs app over a 12-week study period; (2) to assess the impact of the PolarUs app on QoL, as assessed by our primary outcome measure, the QoL.BD; and (3) to leverage mixed qualitative and quantitative methods to provide deeper insights into engagement patterns associated with the PolarUs app and subjective experiences of app use. The remainder of this discussion is divided into 3 major focus areas. The first section discusses our definitions of app use and engagement and the associated theoretical issues. The second section examines the strengths and limitations of this protocol. The third section discusses implications of this protocol. The discussion concludes with an overview of our next steps.

### Defining App Use and Engagement

The purpose of the PolarUs app is to enhance QoL for people living with BD, and this has shaped the design of the app to include self-management tools and educational content; these app design features are also of interest in defining the user engagement metrics for this study. User engagement is the cognitive, temporal, affective, and behavioral investment a person makes when interacting with a digital system [75] and is a foundational element supporting the efficacy of mental health apps [85]. The quality and impact of this investment must be assessed “in relation to the purpose of a particular intervention, and can only be established empirically, in the context of that intervention” [86]. Many digital health interventions focus solely on behavioral engagement *(*eg, frequency and duration of app use or specific app feature use) [87]. Computing the number of log-ins, pages, modules, or features that have been accessed and the length of time spent on these components are commonly used as proxies of engagement breadth and depth [88], but do not consider individual differences in user expectations and use patterns or the dynamic needs of people managing a mental health condition; for example, mood fluctuations and stage of illness [59].

To appreciate the extent to which the PolarUs app is aligned with self-management goals of users [86,89] and clinically relevant outcomes [90], we will analyze use data alongside questionnaire and interview data. The use of mixed methods will allow us to explore engagement trajectories and determine effective use benchmarks by examining behavioral measures with users’ subjective experiences and insights and clinical outcomes. This study design will advance our understanding of the relationship between behavioral and self-report data to deepen our understanding of patterns of engagement with the PolarUs app in particular and mHealth apps in general.

### Limitations

There are several notable limitations to this protocol, the first of which relates to the generalizability of the findings from the study. First, as the PolarUs app is currently only available in English, this will restrict the study to participants with English language skills, thus limiting the generalizability of the findings. To address this limitation, the PolarUs app will soon support Canada’s second national language, French. Indeed, there is already a validated French version of the QoL.BD [91]. This version of the QoL.BD will allow us to build the PolarUs app to support French initially, with the long-term goal of supporting other languages.

Second, it is likely that people with a specific interest in mHealth apps and self-management strategies for BD will self-select into this study. Although this limitation cannot be addressed in this study, future studies could use a more general recruitment strategy, such as advertising a study for self-management strategies for BD in general, rather than for a self-management app for BD. Such a recruitment strategy would also support a study that could compare the efficacy of the PolarUs app to more traditional ways of learning about and using self-management strategies (eg, browser-based psychoeducation, such as the Bipolar Wellness Centre [29]). Future research may also address a possible bias for higher levels of digital health literacy in this study.

Although we aimed to make the PolarUs app as accessible as possible through community consultation, consideration of accessibility issues specific to serious mental health during the design phase [92], and attention to reading level in content writing, it is still likely that individuals with more familiarity with apps and computers will be better able to engage with the intervention and complete all required research tasks (ie, web-based questionnaires) [93]. Although it is not feasible for us to offer dedicated support to upskill participants in technological abilities in the context of this study, future research may evaluate whether adjunctive interventions can enhance the feasibility of mHealth interventions in populations with BD.

A third limitation relates to the purposive sampling strategy used to select participants for qualitative interviews, as this selection process may be subject to researcher bias. Probability-based sampling was considered but ultimately not chosen for reasons of feasibility, given that not all participants will start and conclude the intervention at the same time. Interviewing people as close as possible to the period in which they used the PolarUs app will limit potential recall bias and enhance the depth of information available and will help ensure that our target sample size is achieved. To address the potential bias in our interpretation of engagement with the PolarUs app, we aim to recruit individuals to ensure diversity in levels of engagement with the intervention and demographic characteristics. Further, to ensure transparency, we will report on the demographic characteristics of the qualitative subsample relative to the overall sample.

Finally, it is possible that the user engagement metrics deployed in this study may be influenced by the study design. For example, prior research has shown that user engagement with web- and app-based mental health programs may be influenced by frequent interactions with research assistants [94]. In this study, participants will interact with research assistants at least once a month over the 12-week study period.

### Implications of This Research

This study has several implications. First, in this protocol, we use QoL as the primary outcome variable to determine the impact of our mHealth app. As emphasized previously, QoL is an important outcome variable for any intervention or treatment of a health condition, given that improvements on specific clinical scales do not necessarily translate to tangible improvements in day-to-day life. However, relatively few clinical trials have used QoL outcomes to assess the impact of psychosocial interventions, both in BD and in mental health more generally. It is our hope that assessments of the potential efficacy of mHealth apps via examination of QoL outcomes (combined with more traditional outcomes) will soon become ubiquitous.

Second, the design of the PolarUs app has emphasized co-design with individuals living with BD, clinicians who specialize in the treatment of BD, and BD researchers. The design of this study will allow us to indirectly explore the effectiveness of such community-engaged app design. That is, if the PolarUs app is found to be effective, it will reinforce the notion that mHealth app co-design is critical for the impact of an mHealth app [95]. Future studies should examine which elements of the co-design process are critical for mHealth app efficacy and uptake.

## Abbreviations

BD: bipolar disorder
CREST.BD: Collaborative Research Team to Study Psychosocial Issues in Bipolar Disorder
mHealth: mobile health
QoL: quality of life
QoL.BD: Quality of Life in Bipolar Disorder
UES: User Engagement Scale
WHOQOL-BREF: Brief World Health Organization Quality of Life
